# Diphtheria

**Published:** 1886-03

**Authors:** 


### DIPHTHERIA.

Almost every paper we take up gives an account of the diphtheria and its fatal effects, in different parts of the country. Notwithstanding it has been so many years raging in some parts of the United States and in Europe, there seems to be no discovery that makes it a disease which can be easily controlled. In some instances a doctor states that he has tried a certain remedy and has proved successful; but the next one who tries it fails. So it has generally been found that no remedy is a sure cure for the terrible disease.

But little is said about the diphtheria here, as it is not considered epidemic, but if small-pox or cholera were as prevalent in the city, New York would be shunned by people generally, and thousands would flee to the country from fear of the terrible disease. From August 8th to December 12th last, there were over 400 cases of diphtheria recorded, and probably there were many more; for, as Dr. Nagle states, "not nearly all those who have diphtheria in the city are ever heard of at the Board of Health, unless they die." So that if we place the number having the diphtheria at five hundred, we shall probably be nearer the truth. That is within a part of five months, making the yearly deaths about a thousand. We can imagine what would be the excitement if a thousand cases of small pox or cholera should occur in the same time, yet here is a disease which is absolutely more fatal than Asiatic Cholera stalking forth in our midst, of which we make little note. It is, of course, because the disease does not generally take grown people. It is mostly confined to the young children, but it is nevertheless human beings that are taken away by the dread disease, for which there seems to be no absolute remedy. The efforts made to prevent it is the one thing that seems to be the most sensible thus far discovered to fight it with.

We are acquainted with a physician of long practice in a city of some twenty thousand inhabitants, who pays attention to cases in which he is called and studies them. Some years ago he delivered an address before the Pennsylvania State Medical Society, on the subject of diphtheria. He had not lost a case for years, and he has not, so far as we know, yet lost one, and always follows the same treatment. Other physicians in that vicinity generally give the same remedy, but most of them fear to give as much as the cases require and so often fail. Those in Philadelphia that follow his treatment as to quantity as well as kind of medicine, are generally as successful as he has been. So far as we have been able to discover, it has proved to be an effectual remedy for the complaint.

It is *An Aqueous, Saturated Solution of Chlorate of Potasia.* It is readily taken without admixture. The dose for an adult is a large table-spoonfull every hour, until the force of the disease has abated, when it may be given less frequently for one or two days. It need not be kept up after a day or two if the improvement has commenced. There is no danger from taking the admixture; the blood may become thinner from long use, but generally recovers on a suspension of the medicine.

We have known families, in time of the prevalence of the disease to keep a bottle of the solution on hand all the time; we have known them to have their children take a dose of the medicine occasionally, and we have never known one to be seriously ill with d phtheria who pursued this course. Physicians generally are afraid to give the heroic dose; but the case is one in which it is needed, and there is no kind of danger from. We advise all families, in time of this disease, to keep it on hand, if in cool weather (it would not keep in warm weather), and in warm weather always to have enough in the house to commence treatment on short notice. It will most assuredly be one of the best protections that can be resorted to, and is better than most doctors.

